# Supplementary material for: Apamin Enhances Neurite Outgrowth and Regeneration after Laceration Injury in Cortical Neurons
Source: Toxins (Basel). 2021 Aug 28;13(9):603. doi: 10.3390/toxins13090603 (PMC8472698; doi:10.3390/toxins13090603)
Supplement: Supplementary file 1 [file toxins-13-00603-s001.zip › toxins-1327124-supplementary.pdf]

toxins

Supplementary Materials: Apamin Enhances Neurite Outgrowth and Regeneration after Laceration Injury in Cortical Neurons

Hyunseong Kim, Jin Young Hong, Junseon Lee, Wan-Jin Jeon and In-Hyuk Ha *

Jaseng Spine and Joint Research Institute, Jaseng Medical Foundation, Seoul 135-896, Korea; biology4005@gmail.com (H.K.); vrt3757@gmail.com (J.Y.H.); excikind@gmail.com (J.L.); poghkl@gmail.com (W.-J.J.)

* Correspondence: hanihata@gmail.com; Tel.: +82-2-2222-2740; Fax: +82-2-527-1869

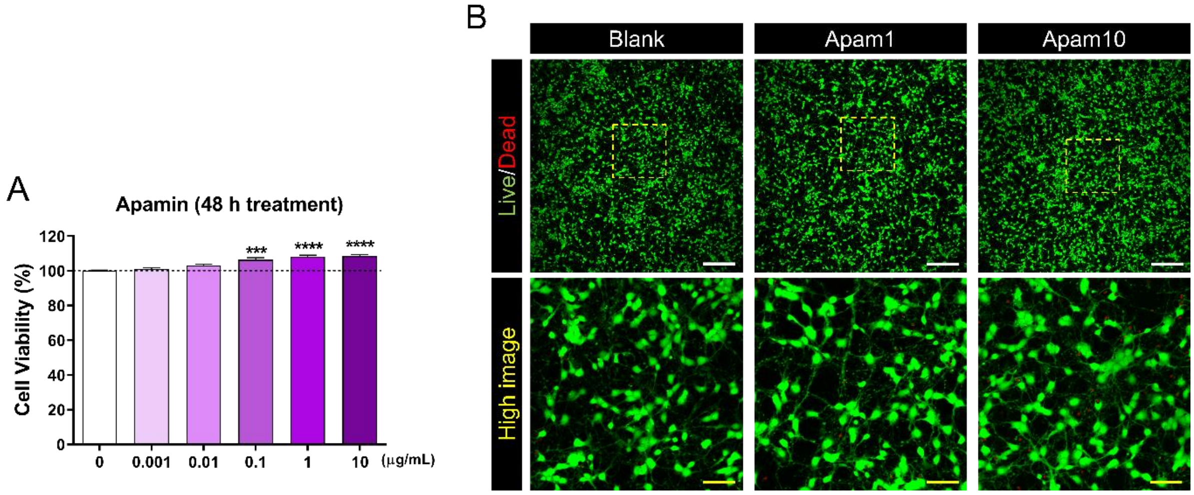

Figure S1. Cell viability assay and live/dead assay after 48 h of the apamin treatment on DIV6 mature cortical neurons. (**A**) CCK assay of neuronal cell viability after 48 h of apamin treatment on DIV6 mature cortical neurons. (**B**) Representative live/dead assay images showing live (green) and dead (red) cells in DIV6 mature cortical neurons with 0.001, 0.01, 0.1, 1, and 10 μg/mL of apamin. White scale bar = 200 μm. Yellow scale bar = 50 μm. Data are expressed as the mean ± standard error of the mean (SEM). Significant differences indicated as *** $p < 0.001$ and **** $p < 0.0001$ vs. the blank group, as analyzed by the one-way analysis of variance (ANOVA) and Tukey's post-hoc test.

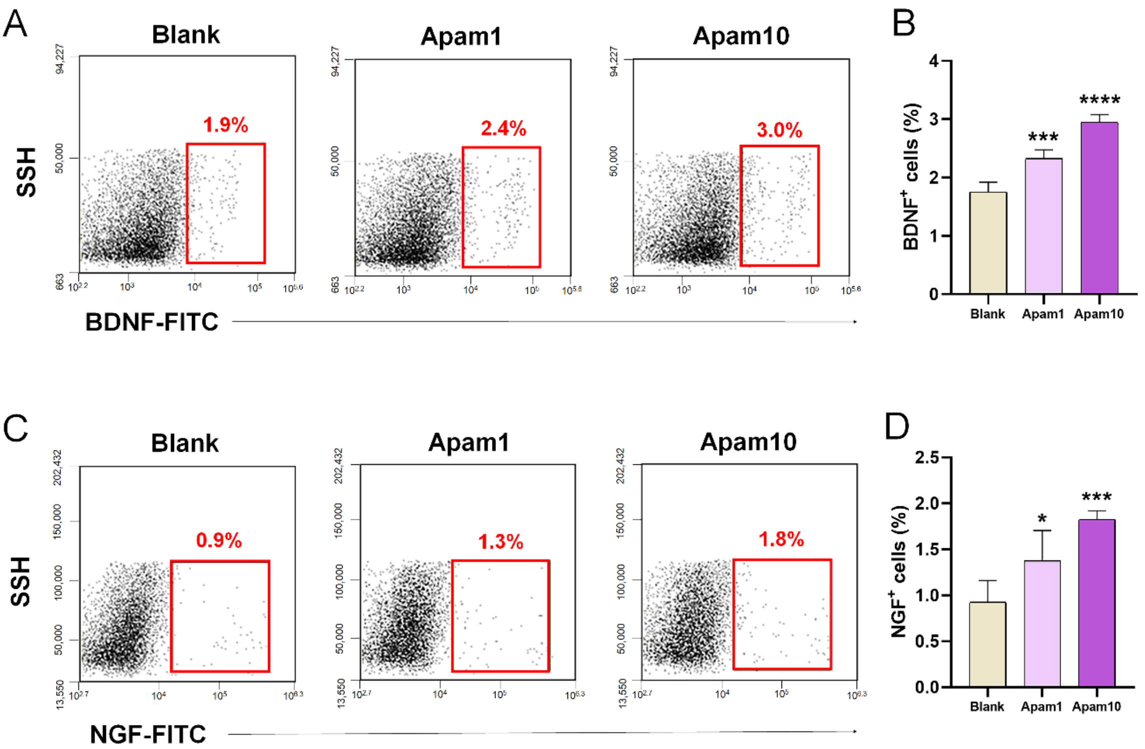

Figure S2. BDNF and NGF flow cytometry analysis of mature cortical neuron treated with 1 and 10 µg/mL of apamin. (**A**) Representative dot plots showing BDNF expression. (**B**) Quantification of BDNF-positive cells at 10,000 single cell events collected for analysis gate. (**C**) Representative dot plots showing NGF expression. (**D**) Quantification of NGF-positive cells at 10,000 events. Data are expressed as the mean ± standard error of the mean (SEM). Significant differences indicated as * $p < 0.05$, *** $p < 0.001$ and **** $p < 0.0001$ vs. the blank group, as analyzed by the one-way analysis of variance (ANOVA) and Tukey's post-hoc test.
